# Clinical features, treatment, and outcomes of celiac-associated arthritis: a retrospective cohort study

**DOI:** 10.1186/s12969-023-00822-x

**Published:** 2023-05-02

**Authors:** Ana Luiza Altaffer, Pamela Weiss

**Affiliations:** 1 Department of Pediatrics, Children’s Hospital of Philadelphia, Philadelphia, PA USA; 2 Department of Pediatrics, Division of Rheumatology and Clinical Futures, Children’s Hospital of Philadelphia and Center for Clinical Epidemiology and Biostatistics, Perelman School of Medicine, University of Pennsylvania, Philadelphia, PA USA; 3 Department of Pediatrics, Division of Rheumatology, Baylor College of Medicine and Texas Children’s Hospital, 1102 Bates Ave Suite 330, Houston, 77030 TX USA

**Keywords:** Arthritis, Celiac disease, Gluten-free diet, Children

## Abstract

**Background:**

Although arthritis is recognized as an extra-intestinal manifestation of celiac disease, little is known about the clinical course and outcomes of pediatric celiac-associated arthritis. This study describes clinical characteristics, treatments, and outcomes of children with celiac-associated arthritis.

**Methods:**

This was a retrospective cohort study of children with celiac disease seen in pediatric rheumatology clinic between 2004 and 2021 for joint complaints. Data was abstracted from electronic health records. Patient demographics and clinical manifestations were evaluated using standard descriptive statistics. Physician- and patient-reported outcomes were evaluated at the index visit, 6-month follow-up, and last recorded visit, and were compared using Wilcoxon signed-rank tests.

**Results:**

Twenty-nine patients with celiac disease were evaluated for joint complaints, and 13 were diagnosed with arthritis. Their mean age was 8.9 years (SD 5.9), and 61.5% were female. Celiac disease diagnosis was made before arthritis diagnosis in only 2 cases (15.4%). Initial testing that led to the celiac disease diagnosis was obtained by the rheumatologist in 6 cases (46.2%). Only 8 patients (61.5%) had concomitant GI symptoms, and of these, 3 patients had BMI z-scores <-1.64 and 1 had impaired linear growth. Arthritis presentation was most often oligoarticular (76.9%) and asymmetric (84.6%). Most cases required systemic therapy (n = 11, 84.6%) with DMARDs, biologics, or both. Of the 10 patients who required systemic therapy and reported compliance with the gluten-free diet, 3 (30%) were able to stop systemic medications. Two of 3 patients who cleared celiac serologies came off systemic medications. Statistically significant improvement was noted in the number of joints involved (p = 0.02) and physician global assessment of disease activity (p = 0.03) between the index and final visit.

**Conclusions:**

Rheumatologists play an important role in the identification of celiac disease, as arthritis was the presenting symptom in most cases and was not always associated with GI symptoms or poor growth. The arthritis was most often oligoarticular and asymmetric. Most children required systemic therapy. The gluten-free diet may not be sufficient to manage arthritis, but antibody clearance may be an indicator of higher likelihood of disease control off medications. Outcomes are promising with a combination of diet and medical therapy.

## Background

Celiac disease is an autoimmune intestinal enteropathy that is triggered by exposure to gluten in genetically susceptible individuals [1]. The prevalence of celiac disease among children is estimated to be around 0.9% [2], and recent studies have noted increasing incidence in many industrialized nations that cannot be fully explained by advances in diagnostics [3]. While celiac disease classically presents with gastrointestinal symptoms and poor growth in children, it is recognized that extraintestinal manifestations, including arthralgia and arthritis, are common and may present in isolation [4]. In one study, joint symptoms were present in 6% of children with celiac disease [5]. Celiac disease is also associated with an increased prevalence of autoimmune diseases, with one study of both pediatric and adult patients reporting a cumulative risk of autoimmune disease to be 8.1% +/- 1% at age 15 and 15.7% +/- 1.5% at age 30. Type 1 diabetes and autoimmune thyroiditis were the most frequently observed, accounting for 29% and 25% of cases respectively [6].

There is also evidence to suggest that the prevalence of celiac disease is higher among children with a diagnosis of juvenile idiopathic arthritis (JIA) than the general pediatric population, with one study citing a prevalence of 2.6% [7]. A recent study found that in comparison to children with JIA and no celiac disease, children with both arthritis and celiac disease tended to have a more severe JIA disease course, requiring systemic medications and step-up therapy despite adoption of a gluten-free diet [8].

Interestingly, the same does not hold true in the adult population [9]. While overlap has been noted between rheumatoid arthritis and celiac disease in terms of epidemiology, pathophysiology, and clinical presentation [10], studies have not demonstrated a clear association between the conditions. In a study of 160 adult patients with seropositive rheumatoid arthritis in New Zealand, the prevalence of celiac disease was not significantly different from that in the general population [11]. In another cross-sectional questionnaire-based study of 356 adult patients with celiac disease and 234 controls, there was no significant difference in reports of arthritic symptoms between the two groups.

While there is evidence to suggest that there is an underlying association between celiac disease and arthritis in children, and that their co-occurrence can have significant impacts to their health and quality of life, much is still unknown about the typical presentation and disease course of pediatric celiac-associated arthritis. The purpose of this study was therefore to describe clinical characteristics, treatment regimens, and outcomes of children with celiac-associated arthritis.

## Methods

This study was reviewed and determined exempt by the Children’s Hospital of Philadelphia (CHOP) Institutional Review Board (IRB 19-016905).

### Patients

The source population for the study was children less than 21-years-old at initial rheumatology evaluation between Sept 2004 and Dec 2021. All patients were diagnosed with celiac disease (ICD-10 K90.0 or ICD-9 579.0) and arthritis (CD-10 M08.xx or ICD-9 714.x, 696.x, 720.x, 721.9x). Patients with presumed celiac disease or gluten intolerance without confirmatory serologic testing or biopsies were excluded. The index visit was defined as the date of initial evaluation by rheumatology.

### Data Collection

Demographics and clinical characteristics were abstracted from the electronic health record. Patient demographic data were abstracted for all patients and included: age at initial rheumatology evaluation, sex, race, ethnicity, BMI z-score, family history of celiac disease, and family history of other autoimmune diseases.

For each patient, the number and type of affected joints, physician assessment of disease activity (visual analogue scale [VAS] 0–10, 0 = best, 10 = worst), patient/caregiver assessment of overall well-being (VAS, 0 = best, 10 = worst), patient/caregiver pain score (VAS, 0 = best, 10 = worst), and clinical juvenile arthritis disease activity score (cJADAS) were recorded at the rheumatology index evaluation (baseline), at the 6 month follow-up (+/- 3 months), and at the last recorded visit. The cJADAS was defined as the sum of the physician assessment of disease activity, patient/parent assessment of overall well-being, and number of affected joints with a maximum of 10 [12]. Additional clinical measures included presence of GI symptoms, impaired linear growth (defined as height z-score less than − 1.64, or less than 5th percentile), timing of celiac diagnosis relative to arthritis diagnosis, specialty of the provider who made the celiac diagnosis, and laboratory data, including antinuclear antibodies (ANA), rheumatoid factor (RF), human leukocyte antigen B27 (HLA-B27), erythrocyte sedimentation rate (ESR), c-reactive protein (CRP), anti-tissue transglutaminase IgA (anti-TTG IgA), anti-tissue transglutaminase IgG (anti-TTG IgG), anti-endomysial antibody (EMA), anti-deamidated gliadin IgA (DGP IgA), and anti-deamidated gliadin IgG (DGP IgG).

Treatment data was collected for patients with arthritis at all of their recorded rheumatology visits and included use of gluten-free diet, nonsteroidal anti-inflammatory drugs, intra-articular corticosteroid injections, disease-modifying antirheumatic drugs (DMARDs), and biologic drugs.

### Statistical Analysis

Demographics and clinical manifestations were evaluated using standard descriptive statistics. Differences in physician- and patient-reported outcomes between initial and last follow-up evaluations were compared using Wilcoxon signed-rank tests.

## Results

### Patients

A total of 29 patients with celiac disease were evaluated in rheumatology clinic for joint complaints. Thirteen (44.8%) were diagnosed with arthritis and 16 (55.2%) had arthralgia but no evidence of active arthritis. The median time from the first to the last recorded rheumatology visit for patients with arthritis was 46 months (IQR 16.5–78.5).

Patient demographics and clinical characteristics at presentation are shown in Table 1. The average age at the index visit was younger in patients with arthritis versus arthralgia only (8.9 vs. 12.6 years). Both groups were predominantly female, white, and not Hispanic or Latino, consistent with prior observations of increased CD prevalence in non-Hispanic white patients [13]. No patients self-identified as American Indian or Alaska Native, Asian, Black or African American, or Native Hawaiian or Other Pacific Islander. About 80% of all patients reported a family history of autoimmune disease, with the most frequent including psoriasis (31.0%), rheumatoid arthritis (27.6%), inflammatory bowel disease (17.2%), systemic lupus erythematosus (13.8%), and Hashimoto thyroiditis (10.3%). Approximately 1/3 also reported a family history of celiac disease.

**Table 1:** Demographics and clinical characteristics

	All patients	Patients with arthritis	Patients with arthralgia
**N**	29	13 (44.8)	16 (55.2)
**Sex n, (%)**			
Male	7 (24.1)	5 (38.5)	2 (12.5)
Female	22 (75.9)	8 (61.5)	14 (87.5)
**Age at rheumatology presentation in years, mean (SD)**	10.9 (4.8)	8.9 (5.9)	12.6 (3.0)
**Race, n (%)**			
White	26 (89.7)	10 (76.9)	16 (100)
Other	4 (13.8)	4 (30.8)	0 (0)
**Ethnicity, n (%)**			
Hispanic or Latino	4 (13.8)	3 (23.1)	1 (6.3)
Not Hispanic or Latino	25 (86.2	10 (76.9)	15 (93.8)
**BMI z-score at rheumatology presentation, mean (SD)**	-0.25 (1.18)	-0.85 (1.09)	0.20 (1.08)
**Family history of celiac disease, n (%)**	10 (34.5)	5 (38.5)	5 (31.3)
First-degree relative	7 (24.1)	3 (23.1)	4 (25.0)
Second-degree relative	2 (6.9)	1 (7.7)	1 (6.3)
Third-degree relative	1 (3.4)	1 (7.7)	0 (0)
**Family history of other autoimmune disease, n (%)**	23 (79.3)	11 (84.6)	12 (75.0)

*Legend.* ^*a*^ *11 patients tested.*

Of the patients with arthritis, 30.8% and 7.7% were positive for ANA and RF, respectively. Of the 11 tested for HLA-B27, 1 (9.1%) was positive. Whereas ESR was normal at presentation in most patients (76.9%), CRP was elevated in 8 (61.5%) patients (> 2x normal in 5 patients and 1-2x normal in 3 patients). One patient was already on a gluten-free diet at the time of rheumatology presentation. Only one patient had concomitant uveitis, and that patient was ANA negative.

### Characteristics of joint involvement

On average the diagnosis of arthritis occurred 4.7 months (SD 3.6) after the onset of joint symptoms. The presentation was most commonly oligoarticular (76.9%) and asymmetric (84.6%). The median number of active joints at presentation was 2 (IQR 1–5). The most frequently involved joint was the knee (n = 9, 69.2%), and knee involvement was most often unilateral. Other commonly affected joints are listed in Table 2 and included ankles (n = 4, 30.8%), finger PIPs (n = 3, 23.1%), and wrists (n = 3, 23.1%).

**Table 2:** Joints with arthritis at index visit

Joint	Patients with joint involvement, n (%)	Unilateral, n (%)	Bilateral, n (%)
**Knee**	9 (69.2)	7 (77.8)	2 (22.2)
**Ankle**	4 (30.8)	4 (100)	0 (0)
**Finger PIPs**	3 (23.1)	0 (0)	3 (100)
**Wrist**	3 (23.1)	1 (33.3)	2 (66.7)
**Elbow**	1 (7.7)	0 (0)	1 (100)
**Finger MCPs**	1 (7.7)	1 (100)	0 (0)
**Subtalar**	1 (7.7)	1 (100)	0 (0)
**Toe DIPs**	1 (7.7)	1 (100)	0 (0)
**Toe PIPs**	1 (7.7)	1 (100)	0 (0)

Of the patients who had arthralgia without arthritis, the mean number of tender joints was 4.6 (SD 4.9). Patients most often reported large joint arthralgia. The knee was the most common tender joint (62.5%), followed by the ankle (50.0%), shoulder (25.0%), and hip (25.0%). Patients also frequently reported non-articular pain, including generalized body pain (n = 3), shin pain (n = 2), arm pain (n = 1), and pain involving feet, hands and/or digits (n = 5).

### Celiac disease features among patients with arthritis

Celiac disease features are summarized in Table 3. The celiac disease diagnosis was made prior to the arthritis diagnosis in only 2 cases (15.4%). The timing of celiac diagnosis ranged from 51 months prior to 59 months after the arthritis diagnosis, with a median of 3 months following the arthritis diagnosis (IQR 0–19). Initial celiac testing that led to the diagnosis was obtained by the patient’s rheumatologist in 6 (46.2%) cases, by a gastroenterologist in 4 (30.8%) cases, by the PCP in 2 (15.4%) cases, and by an endocrinologist in 1 (7.7%) case. Of the 10 patients who were not IgA deficient, all had an elevated TTG IgA. Of the three that were IgA deficient, two had TTG IgG testing, and one was positive. All but one of the cases were biopsy-confirmed. The single case that was not biopsy-confirmed was due to family preference to adopt a gluten-free diet based on laboratory testing without endoscopy. Eight patients (61.5%) reported concomitant GI symptoms. Of these, only 3 patients had a BMI z-score less than − 1.64, and only one patient had impaired linear growth.

**Table 3:** Celiac Disease Features

	Patients with arthritis N = 13
**Timing of celiac disease diagnosis relative to arthritis diagnosis in months, median (IQR)**	3 (0–19)
**Provider who initiated CD testing, n (%)**	
Rheumatologist	6 (46.2)
Gastroenterologist	4 (30.8)
PCP	2 (15.4)
Endocrinologist	1 (7.7)
**Biopsy-confirmed diagnosis, n (%)**	12 (92.3)
**Laboratory testing at celiac diagnosis, n (%)**	
IgA deficient	3 (23.1)
Elevated TTG IgA, not IgA deficient	10 (100.0)
Elevated TTG IgG, IgA deficient	1 (50.0)^a^
Elevated EMA	9 (75.0)^b^
Elevated DGP IgA	6 (60.0)^c^
Elevated DGP IgG	5 (62.5)^d^
**Patients with concomitant GI symptoms, n (%)**	8 (61.5)
**Patients with concomitant impaired linear growth**^**e**^, **n (%)**	1 (7.7)
**Underweight**^**f**^ **patients, n (%)**	3 (23.1)

*Legend.* ^*a*^ *2 patients tested.*

^*b*^ *12 patients tested.*

^*c*^ *10 patients tested.*

^*d*^ *8 patients tested.*

^*e*^ *Impaired linear growth defined as height z-score less than − 1.64, or bottom 5%.*

^*f*^ *Underweight defined as BMI z-score less than − 1.64, or bottom 5%.*

### Treatment

Treatments received over the course of therapy for celiac-associated arthritis are demonstrated in Table 4.

**Table 4:** Treatments received over course of therapy for celiac-associated arthritis

Treatment	n (%)	Time to start in months*, median (IQR)
**GF diet**	13 (100)	7 (1–17)
**Intra-articular corticosteroid injections**	10 (76.9)	0 (0–1)
**DMARD** Methotrexate Hydroxychloroquine Sulfasalazine	10 (76.9) 10 (100) 1 (10.0) 1 (10.0)	8 (2–19) 8 (2–19) 11 (n/a) 47 (n/a)
**Biologic** Adalimumab Etanercept Tocilizumab Secukinumab Rituximab	7 (53.8) 6 (85.7) 2 (28.6) 1 (14.3) 1 (14.3) 1 (14.3)	13 (1-138) 9.5 (5–27) 69.5 (1-138) 38 (n/a) 9 (n/a) 36 (n/a)

Legend. * Time to start treatment, index visit = time 0.

Four of the twelve patients (33.3%) who were seen at a 6-month follow-up visit reported starting a gluten-free diet. However, of those, none had cleared celiac antibodies. The remaining patients did not yet have a confirmed diagnosis of celiac disease. All patients were started on a gluten-free diet once celiac disease diagnosis was confirmed, and all but one reported compliance with the diet at the last documented rheumatology visit. Nevertheless, only 3 patients (23.1%) had cleared celiac antibodies by the last documented visit (median time from celiac diagnosis 64 months, IQR 42–164); clearance was documented a median of 14 months (IQR 3–35) after initiation of the gluten-free diet.

Ten children (76.9%) underwent intra-articular joint injections, eight of which occurred in the first 6 months of treatment. Eleven (84.6%) were started on systemic therapy with a DMARD (n = 4), a biologic (n = 1) or both (n = 6). Seven of these started systemic medications in the first 6 months after diagnosis. Two patients (16.7%) were never on systemic medications and achieved remission with intra-articular corticosteroid injections and gluten-free diet alone. Both patients had mild joint disease from onset, with only one joint involved at diagnosis. One was a 15-year-old male with negative ANA and right wrist arthritis, and the other was a 3-year-old female with positive ANA and right knee arthritis.

Of the 10 patients who were started on systemic medications and reported compliance with the gluten-free diet, 3 (30%) successfully stopped systemic medications. Two of the patients who stopped medications had cleared celiac antibodies. Seven patients (70%) remained on systemic medications at the final visit, despite reported adherence to the gluten-free diet. Of the 7 patients who remained on systemic medications, only 1 had cleared celiac antibodies. Of those, 2 were on methotrexate, 1 was on adalimumab, 3 were on a combination of methotrexate and adalimumab, and one was on methotrexate and had received a rituximab infusion about 1 month prior. The latter was the only patient who had active arthritis at the final visit.

### Disease course

**Fig. 1:**
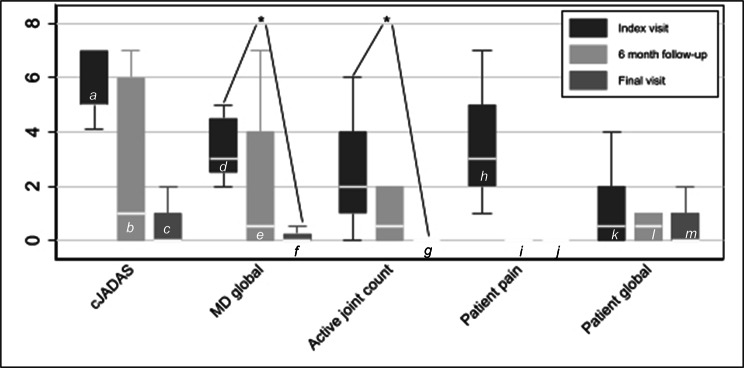
Disease activity and patient-reported outcomes Legend. *p < 0.05 ^*a*^ *5 documented.* ^*b*^ *9 documented.* ^*c*^ *9 documented.* ^*d*^ *8 documented.* ^*e*^ *10 documented.* ^*f*^ *12 documented.* ^*g*^ *12 documented. One patient seen intelemedicine visit and had no joint exam documented.* ^*h*^ *6 documented.* ^*i*^ *9 documented.* ^*j*^ *10 documented.* ^*k*^ *6 documented.* ^*l*^ *9 documented.* ^*m*^ *10 documented.*

Physician and patient-reported measures of disease activity at the baseline visit, at the 6-month follow-up, and at the final recorded visit are demonstrated in Fig. 1.

There was a statistically significant improvement in number of joints with active arthritis (p = 0.02) between the baseline and final visit. Only one patient had active arthritis at the final visit, with 13 joints involved despite adherence to systemic medications. Her underlying diagnosis was in question given her severe presentation despite RF negativity. There was also a statistically significant improvement in physician assessment of disease activity (p = 0.03) between the baseline and final visit. Patient reported outcomes trended towards improvement, but did not achieve statistical significance.

## Discussion

This study describes clinical features, treatment regimens, and outcomes of the largest cohort to date of children with celiac-associated arthritis evaluated in a pediatric rheumatology clinic. Arthritis was the presenting symptom of celiac disease in a majority of cases, with only 2 patients with a pre-existing diagnosis of celiac disease. The diagnostic evaluation for celiac disease was initiated by a rheumatologist in over half of patients. These findings, along with prior evidence of increased prevalence of celiac disease among patients with JIA [7], suggest that rheumatologists play an important role in the identification of celiac disease. Joint involvement was most often oligoarticular and asymmetric, and the knee was the most frequently affected joint. Most children required systemic therapy despite adherence to the gluten-free diet. While few children cleared celiac serologies after starting the gluten-free diet, 2 out of 3 of those children were able to stop medications for arthritis. Lastly, although uveitis was not common, it did affect a single ANA-negative patient.

There is no consensus regarding whether arthritis in children with celiac disease should be termed JIA or celiac-associated arthritis. We have opted to refer to it as celiac-associated arthritis given that it is not truly idiopathic, but recognize that this terminology is not universally agreed upon.

Recently, a study from Italy reported that 8 patients with celiac disease and arthritis had a more severe disease course than patients without celiac disease (n = 321), particularly in regard to an increased need for step-up therapy with a biologic medication in patients with both arthritis and celiac disease. None of the patients were able to discontinue systemic therapy after starting the gluten-free diet [8]. Our study corroborates this finding of need for systemic therapy in a larger group of children.

There is a paucity of clear guidelines for follow-up monitoring of serologies in patients with celiac disease. CD-related antibody titers generally decrease with the gluten-free diet, and changes in antibody levels are associated with degree of compliance. However, these assays do not perform well in distinguishing patients who are partially compliant with the gluten-free diet from those who are fully compliant [14]. Nevertheless, there may still be an important role for monitoring of serologies. A study of 150 children undergoing follow-up biopsy at least 1 year after CD diagnosis demonstrated a high negative predictive value of serologies in assessing for mucosal healing. [15].

While the large proportion of patients who did not clear serologies in our studies is suggestive of incomplete compliance with the gluten-free diet, despite reported compliance, it is impossible to determine compliance with certainty based on these serologies alone. Interestingly, 2 of 3 children from our cohort who cleared celiac antibodies were able to discontinue systemic medications, which suggests that antibody clearance might be an indicator of higher likelihood of success off systemic medications. This warrants further investigation.

Similar to the Italian cohort, we also found uveitis in our cohort of patients with celiac disease, though at a lower prevalence (7.7% versus 25% in the Italian cohort). Since both cohorts are limited in sample size, the prevalence of eye inflammation and determination of whether risk factors for eye disease are the same as for children without celiac disease requires further study. Interestingly, our patient with uveitis was ANA negative. Until further studies are conducted, we suggest following JIA uveitis screening guidelines [16] for these patients.

The European Society Paediatric Gastroenterology, Hepatology and Nutrition (ESPGHAN) 2020 celiac diagnosis guidelines recommend testing children with a variety of GI and extraintestinal symptoms and specific conditions for celiac disease. JIA is not one of these conditions. The guidelines highlight chronic or intermittent diarrhea, distended abdomen, failure to thrive, and stunted growth/short stature as particularly common symptoms of celiac disease [17]. It is therefore reasonable to assess for these symptoms when children present to rheumatology for arthritis, and to screen for celiac disease if any of these symptoms are present. However, in our cohort, a large proportion of patients did not have these symptoms. Only 8 children (61.5%) had concomitant GI symptoms, and of these only 3 had low BMI and only 1 had poor linear growth. Symptom-based testing would have missed the diagnosis in over one third of children. This adds to prior literature [7, 18] supporting screening of all children with JIA for celiac disease, even if otherwise asymptomatic.

The case for celiac disease screening in this population is further strengthened by understanding the complications associated with the condition. Several studies in the adult population have found higher rates of malignancy among patients with celiac disease, including lymphomas [19, 20] and small bowel carcinomas [21, 22]. Studies have also demonstrated an increased prevalence of autoimmune diseases in both adults and children with celiac disease [6]. While there is no clear consensus on whether or not a gluten-free diet can help prevent the development of these complications [23], identification of children with celiac disease allows for long-term follow-up and monitoring. This is particularly important in light of recently increasing incidence of celiac disease [3].

There are several limitations of this study including limited sample size and retrospective nature. Despite the limited sample size, this is the largest study of children with arthritis and celiac disease reported to date. Our findings build upon those of the Italian cohort and together add valuable information that is needed to guide the care and inform guidance for this population. Missing data is inherent to retrospective research; however, none of the physical exam or treatment related data was missing on our cohort. Celiac disease is not routinely screened for in our clinic, so our cohort is likely an underrepresentation of disease. However, this should not impact the importance of our findings. Lastly, the study does not capture the full disease course of every patient, as some patients are still being managed in the pediatric rheumatology clinic. This is an understudied area that requires further investigation.

## Conclusions

In conclusion, arthritis can be the presenting symptom of celiac disease, even in the absence of GI symptoms or poor growth. Children should therefore be assessed for additional signs or symptoms suggestive of celiac disease upon arthritis diagnosis, family history of celiac disease should be sought, and screening of all patients with arthritis should be considered. The gluten-free diet may not be sufficient to manage arthritis, but antibody clearance may be an indicator of higher likelihood of disease control off medications. This finding warrants further investigation. Further studies are also needed to assess the prevalence and risk factors for uveitis in this population.

## Data Availability

The datasets used during the current study are available from the corresponding author on reasonable request.

## List of abbreviations

JIA: Juvenile idiopathic arthritis
VAS: Visual analogue scale
cJADAS: Clinical juvenile arthritis disease activity score
ANA: Antinuclear antibodies
RF: Rheumatoid factor
HLA-B27: Human leukocyte antigen B27
ESR: Erythrocyte sedimentation rate
CRP: C-reactive protein
TTG: Tissue transglutaminase
EMA: Anti-endomysial antibody
DGP: Deamidated gliadin
DMARDs: Disease-modifying antirheumatic drugs
ESPGHAN: European Society Paediatric Gastroenterology, Hepatology and Nutrition
